# Functional Validation and Comparison Framework for EIT Lung Imaging

**DOI:** 10.1371/journal.pone.0103045

**Published:** 2014-08-11

**Authors:** Bartłomiej Grychtol, Gunnar Elke, Patrick Meybohm, Norbert Weiler, Inéz Frerichs, Andy Adler

**Affiliations:** 1 Department of Medical Physics in Radiology, German Cancer Research Centre (DKFZ), Heidelberg, Germany; 2 Fraunhofer Project Group for Automation in Medicine and Biotechnology, Mannheim, Germany; 3 Department of Anesthesiology and Intensive Care Medicine, University Medical Center of Schleswig-Holstein, Kiel, Germany; 4 Department of Anesthesiology, Intensive Care Medicine and Pain Therapy, University Hospital Frankfurt, Frankfurt am Main, Germany; 5 Systems and Computer Engineering, Carleton University, Ottawa, Ontario, Canada; University of Catania, Italy

## Abstract

**Introduction:**

Electrical impedance tomography (EIT) is an emerging clinical tool for monitoring ventilation distribution in mechanically ventilated patients, for which many image reconstruction algorithms have been suggested. We propose an experimental framework to assess such algorithms with respect to their ability to correctly represent well-defined physiological changes. We defined a set of clinically relevant ventilation conditions and induced them experimentally in 8 pigs by controlling three ventilator settings (tidal volume, positive end-expiratory pressure and the fraction of inspired oxygen). In this way, large and discrete shifts in global and regional lung air content were elicited.

**Methods:**

We use the framework to compare twelve 2D EIT reconstruction algorithms, including backprojection (the original and still most frequently used algorithm), GREIT (a more recent consensus algorithm for lung imaging), truncated singular value decomposition (TSVD), several variants of the one-step Gauss-Newton approach and two iterative algorithms. We consider the effects of using a 3D finite element model, assuming non-uniform background conductivity, noise modeling, reconstructing for electrode movement, total variation (TV) reconstruction, robust error norms, smoothing priors, and using difference vs. normalized difference data.

**Results and Conclusions:**

Our results indicate that, while variation in appearance of images reconstructed from the same data is not negligible, clinically relevant parameters do not vary considerably among the advanced algorithms. Among the analysed algorithms, several advanced algorithms perform well, while some others are significantly worse. Given its vintage and ad-hoc formulation backprojection works surprisingly well, supporting the validity of previous studies in lung EIT.

## Introduction

Electrical impedance tomography (EIT) has been proposed as a tool to monitor and guide ventilator therapy in mechanically ventilated patients [1]–[4]. Medical interest in EIT is driven by the discovery of the injurious effects of artificial ventilation [5] and, thus, the importance of lung protective ventilation strategies [6], [7]. No consensus exists as to how optimal ventilator settings (i.e. least harmful and still securing proper gas exchange) should be chosen in an individual patient [8]. However, there is a clear need for continuous assessment of regional lung ventilation. No medical examination technique is available at the bedside allowing this type of monitoring. With its inherent advantages of the radiation-free measuring principle, good time resolution, portability and ability to assess rapid changes in regional lung air content, EIT promises to fulfil many of the requested criteria for such patient monitoring.

A growing body of research seeks to develop and evaluate EIT-based parameters to guide ventilator therapy [1]. Most of these studies used versions of the original back-projection algorithm of [9] for image reconstruction; for recent examples see e.g. [10]–[17]. Newer types of image reconstruction procedures allow theoretical advantages [18]–[21]; however, these algorithms have not been systematically compared against each other, and often have not been tested in vivo. This paucity of systematic validation and comparison of EIT algorithms hampers its entrance into clinical practice.

The ability of EIT to measure regional distribution of ventilation has been validated against several established high-resolution imaging modalities like X-ray computed tomography [22], [23], electron beam computed tomography [24], single photon emission computed tomography [25], [26] and positron emission tomography [27].

Such validation of EIT to anatomical references is important, but exhibits certain drawbacks. Most such studies have not used anatomical models for EIT reconstruction (typically using circles on a 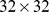 pixel grid) making comparison with morphologically accurate images problematic. Also, the inherent low resolution of EIT creates large partial volume effects [28], while the scan rate of EIT is much higher (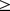
50 frames/s for some devices) than those of the reference radiological techniques. The most important drawback of such studies, however, is that anatomical validation is primarily sensitive to morphological accuracy, and thus less sensitive to the functional performance of EIT imaging. We believe that a functional imaging technique should also have a functional validation methodology. Thus, we propose a more functional approach for validation and comparison of EIT images obtained with different reconstruction algorithms where the ability to provide clinically relevant information is tested directly. Therefore, the aim of our study was to develop a framework to validate and compare EIT images by testing their ability to correctly reflect clinically significant changes in regional lung ventilation (or lack thereof). The backbone of our framework is an experimental EIT data set acquired in mechanically ventilated pigs. By manipulating three clinically relevant ventilator settings – tidal volume (V_T_), peek end-expiratory pressure (PEEP) and the O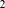 fraction in inspired gas (F_I_ o_2_) – we were able to achieve different regional ventilation distributions following well defined large and discrete shifts in global and regional lung air content.

We used the proposed framework to compare the performance of a number of popular reconstruction algorithms, including GREIT [29] and backprojection. We sampled the analysed algorithms mainly from the large class of sensitivity-based methods available in the EIDORS suite [30]. In doing so, we studied the effect of individual design choices in assembling such algorithms. Other approaches, such as those based on a Bayesian framework (e.g. [31]), monotonicity (e.g. [32]), level sets (e.g. [33], [34]), factorisation (e.g. [35], or the D-Bar method (e.g. [36]) were not evaluated, as they have seen little evaluation for thoracic EIT data (but see [37], [38]. However, we make the data and software publicly available to allow extending the comparison to a wider range of present and future algorithms.

## Methods

### Ethics statement

After approval by the Committee for Animal Care of the Christian-Albrechts University Kiel (Permit Number: V742-72241.121-39), the study was conducted in compliance with the recommendations in the Guide for the Care and Use of Laboratory Animals of the National Institutes of Health.

### Evaluation framework

**Figure 1:**
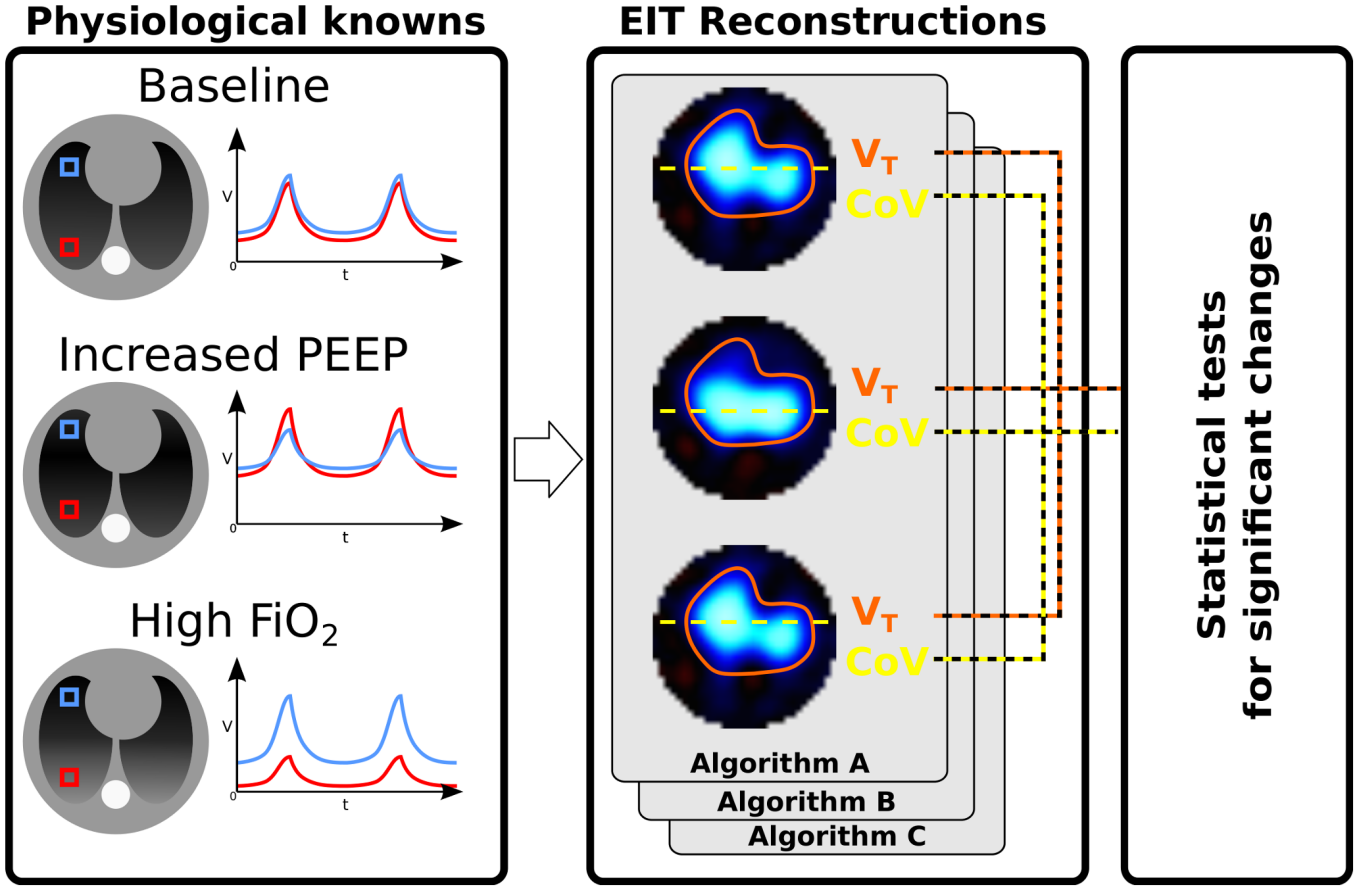
Overview of the proposed methodology. Peep – positive end-expiratory pressure; F_I_ o_2_ - fraction of oxygen in inspired gas; V – air volume; t – time; V_T_ – tidal volume; CoV - centre of ventilation; FEM – finite element model.

Since the potential role of EIT in ventilation therapy is to detect changes in the regional distribution of ventilation, our proposed validation framework is explicitly designed to test its ability to do so. An overview of the framework is presented in Fig. 1.

First, we conducted an experiment in healthy pigs ventilated mechanically to produce shifts in ventilation distribution while keeping V_T_ constant. Ventilation with pure O_2_ is known to induce absorption atelectasis in the dependent, i.e. posterior in supine animals, lung regions [39], [40], while Peep leads to a redistribution of inspired V_T_ with more gas entering the dependent lung regions [41]. These well-known effects of changing Peep and F_I_ o_2_ settings allowed the definition of distinct changes in global and regional ventilation, which are summarized in Table 1.

**Table 1:** Expected effects of changes in ventilation therapy.

Intervention	Tidal volume	Ventilation distribution
Increase in Peep	no change	increased ventilation in dependent lung areas
100% F_I_ o_2_	no change	decreased ventilation in dependent lung areas
Return to baseline	no change	return to baseline

Peep, positive end-expiratory pressure; F_I_ o_2_, fraction of O_2_ in inspired gas.

Second, EIT-derived measures of V_T_ and distribution of ventilation along the gravitational axis were calculated for each analysed algorithm for each animal and condition. These values were used to test each algorithm by comparing against the expected findings, or physiological references (Table 1). For example, V_T_ is a known volume change in the lung. While the distribution of V_T_ changes with Peep, the volume is fixed. Thus, using this physiological constant, we can test that an EIT algorithm which is equally sensitive to volume across the image will, correctly, identify V_T_ as constant, while an EIT algorithm which has spatially varying volume response, may see varying V_T_. Similarly, the known movement of V_T_ toward dependent lung with increases in Peep can be used to test the sensitivity of EIT algorithms to movement within the image. The complete list of physiological references is given in Table 4.

**Table 4:** Performance of different image reconstruction algorithms during mechanical ventilation with constant tidal volume and variable end-expiratory pressure and O_2_ fraction.

Expected finding	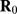	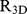	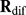	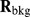	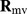	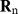	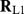	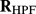	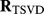	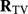	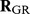	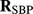
V 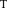 **is independent of** P**eep**												
V 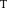 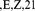 = V 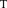 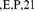	0.224	0.268	0.512	0.838	0.024*	0.008*	0.000*	0.225	0.247	0.815	0.018*	0.044*
V 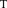 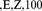 = V 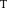 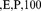	0.022*	0.027*	0.090	0.247	0.008*	0.014*	0.000*	0.026*	0.030*	0.245	0.010*	0.013*
V 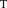 **is independent of** F_I_ o_2_												
V 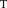 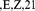 = V 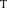 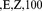	0.010*	0.009*	0.000*	0.010*	0.033*	0.025*	0.000*	0.009*	0.035*	0.030*	0.010*	0.004*
V 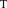 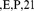 = V 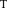 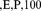	0.002*	0.002*	0.010*	0.005*	0.001*	0.001*	0.000*	0.002*	0.001*	0.042*	0.002*	0.001*
V 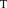 **is reproducible**												
V 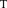 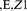 = V 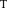 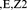	0.003*	0.003*	0.001*	0.012*	0.002*	0.001*	0.000*	0.002*	0.003*	0.007*	0.001*	0.001*
V 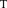 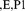 = V 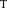 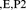	0.000*	0.000*	0.001*	0.002*	0.001*	0.001*	0.000*	0.000*	0.001*	0.002*	0.001*	0.000*
**CoV is** P**eep** **dependent**												
CoV 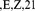 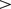 CoV 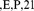	0.088	0.088	0.136	0.114	0.060	0.037*	0.121	0.085	0.075	0.083	0.043*	0.064
CoV 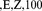 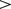 CoV 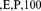	0.034*	0.033*	0.044*	0.052	0.026*	0.017*	0.053	0.033*	0.035*	0.022*	0.019*	0.023*
**CoV is** F_I_ o_2_ **dependent**												
CoV 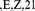 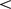 CoV 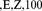	0.012*	0.012*	0.020*	0.020*	0.010*	0.010*	0.035*	0.011*	0.016*	0.009*	0.011*	0.010*
CoV 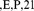 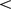 CoV 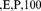	0.659	0.679	0.689	0.713	0.586	0.639	0.751	0.662	0.664	0.735	0.604	0.683
**CoV is reproducible**												
CoV 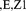 = CoV 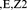	0.059	0.064	0.238	0.013*	0.061	0.023*	0.009*	0.065	0.107	0.026*	0.032*	0.007*
CoV 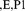 = CoV 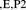	0.009*	0.009*	0.010*	0.000*	0.028*	0.016*	0.001*	0.010*	0.042*	0.006*	0.017*	0.002*

The expected physiological findings regarding the magnitude and distribution of ventilation are used as reference states. 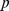-values of the Student's 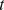-test are reported. For each test, 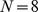 (the number of animals).

E, Measures by EIT; V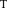, tidal volume; Peep (P), positive end-expiratory pressure; ZEEP (Z), zero end-expiratory pressure; F_I_ o_2_, fraction of O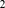 in inspired gas; 21, F_I_ o_2_ equal to 21%; 100, F_I_ o_2_ equal to 100%; indices 1 and 2 identify the first and the second measurements at identical ZEEP or Peep levels; CoV, center of ventilation; 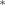 hypothesis confirmed at 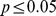.

#### Experimental protocol

The study was performed at the University Medical Center of Schleswig-Holstein, Campus Kiel in Germany. The experiments were carried out on eight anesthetized pigs (body weight (bw): 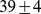 kg, mean
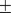
standard deviation (SD)) in supine position. The animals were at first sedated with azaperon (8 mg/kg bw). Anaesthesia was achieved by a continuous intravenous infusion of propofol (6 to 12 mg/kg bw per hour) and sufentanile (10 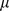
g/kg bw per hour). Subsequently, the animals were intubated and connected to a ventilator (Siemens Servo 900 C ventilator, Siemens-Elema, Solna, Sweden). Haemodynamic as well as ventilatory parameters including heart rate, partial pressure of CO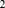 (Pco_2_) in respired gas, arterial O_2_ saturation (S_a_ o_2_), airway pressures and respiratory system compliance were continuously monitored using the S/5 anaesthesia monitoring system with a gas-density compensated module (M-CAIOV, Datex Ohmeda, Helsinki, Finland). All animals were ventilated in a volume-controlled mode with a constant V_T_, respiratory rate (20 breaths/min) and inspiration-to-expiration ratio of 1∶2 in order to maintain normocapnia (end-tidal Pco_2_ 35–45 mmHg). If required, vecuronium bromide (0.1 mg/kg bw) was administered for muscle paralysis to suppress spontaneous breathing activity and thus avoid disturbance of the volume-controlled ventilation measurements.

During the examination period of 60 min, the animals were ventilated with different combinations of end-expiratory pressures and F_I_ o_2_ (Table 2). F_I_ o_2_ was consecutively changed from 21% to 100% and back to 21% and at each F_I_ o_2_, the animals were at first ventilated with zero end-expiratory pressure (ZEEP) and then with Peep of 5 cmH_2_O. In each condition, one EIT measurement was performed meaning that a total of six measurements was obtained in each animal.

**Table 2:** Lung function and ventilation parameters  SD during study period.

Stage	I	II	III	IV	V	VI
Time period, min	0–15	15–20	20–35	35–40	40–55	55–60
Peep, cmH_2_O	0	5	0	5	0	5
F_I_ o_2_, %	21	21	100	100	21	21
V 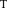 , ml/kg bw	10.1 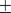 1.6	9.9 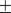 1.7	10.3 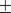 1.8	10.0 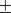 1.7	9.9 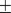 1.5	9.9 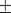 1.7
P 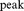 , cmH_2_O	21 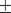 6	23 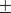 5	20 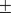 4	23 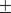 5	20 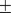 5	23 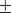 5
P 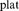 , cmH_2_O	16 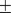 5	20 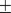 4	15 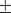 4	19 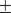 4	16 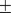 4	20 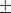 4
C_rs_, ml/cmH_2_O	26.4 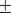 4.2	27.0 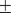 4.5	28.4 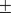 4.8	25.7 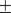 4.0	28.2 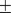 4.2	27.1 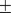 4.0
S_a_ o_2_, %	95 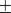 3	97 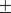 3	98 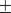 4	98 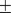 2	97 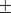 2	96 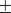 3

Peep, positive end-expiratory pressure; F_I_ o_2_, fraction of O_2_ in inspired gas; V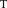, tidal volume; bw, body weight; P_peak_, peak airway pressure; P_plat_, inspiratory plateau airway pressure; C_rs_, respiratory system compliance; S_a_ o_2_, saturation of O_2_.

EIT examinations were performed using the Goe-MF II EIT device (CareFusion, Höchberg, Germany). Sixteen self-adhesive electrodes (Blue Sensor BR-50-K, Ambu, Bad Nauheim, Germany) were placed on the chest circumference in one transverse plane lying approximately at the level of the 6th intercostal space. Electrical currents (50 kHz, 5 mArms) were applied through adjacent pairs of electrodes in a rotating mode. After each current injection, the resulting potential differences were measured by the remaining electrode pairs. EIT data were acquired at a frame rate of 13 images/s during 60 s time intervals.

### EIT algorithms

We review EIT image reconstruction algorithms and develop a taxonomy of approaches. A representative sample of algorithm choices are selected for evaluation.

#### EIT image reconstruction

**Figure 2:**
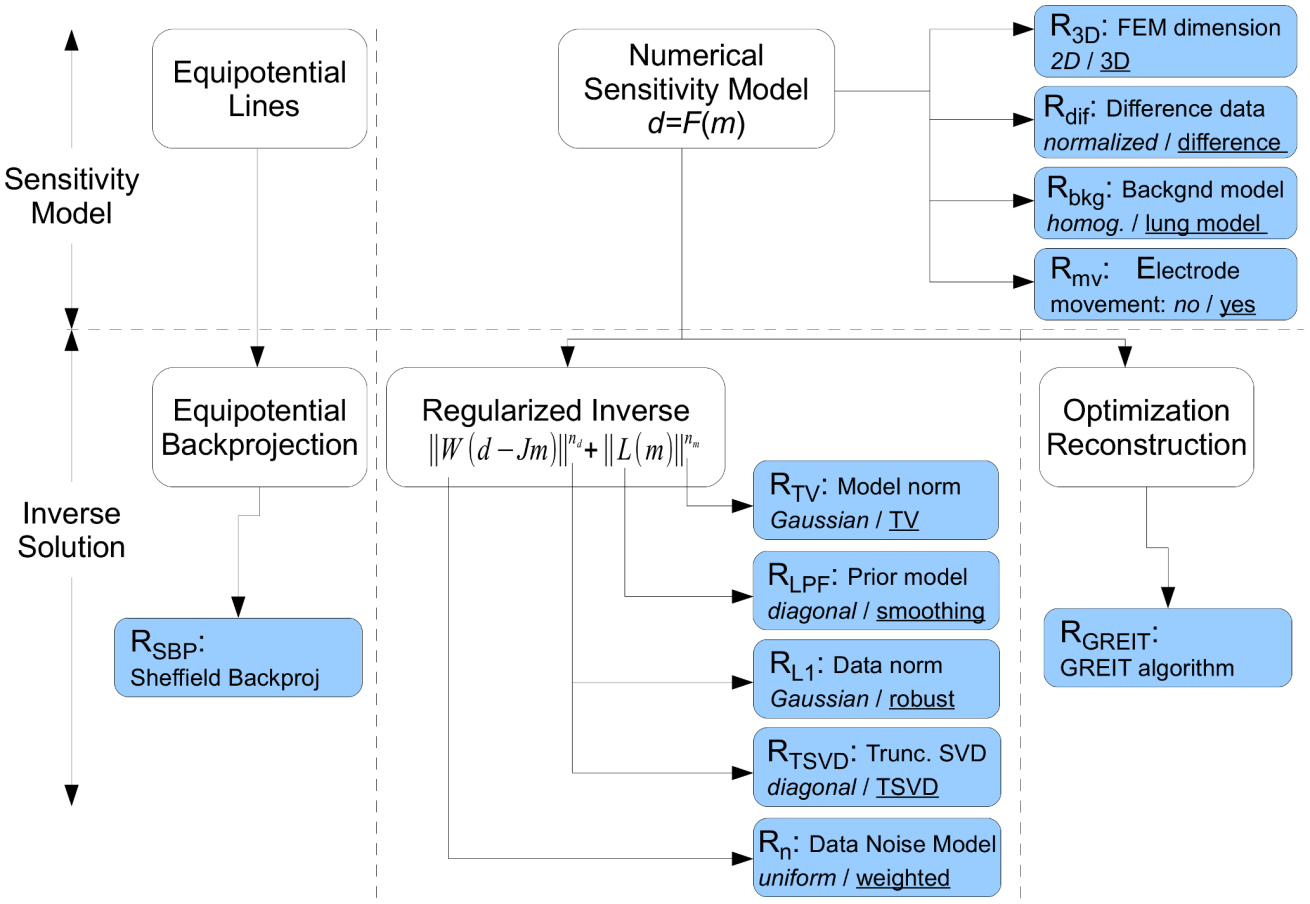
Taxonomy of direct EIT reconstruction algorithms, classified in terms of the selection of forward and inverse model parameters.

The recovery of information about internal conductivity distribution from surface voltage measurements is a severely ill-posed non-linear inverse problem. Time difference EIT (TD-EIT), which compares two sets of measurements, is much less sensitive to uncertainties in electrode placement, thorax geometry and some hardware errors (such as gain variability between channels), than algorithms that attempt to reconstruct the absolute conductivity distribution [42]. This paper considers only TD-EIT algorithms. Specifically, TD-EIT seeks to reconstruct an image of the change in conductivity (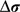) between EIT measurements 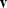 and 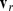, where 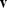 represents the current frame of EIT measurements, while 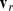 is the reference measurement frame, typically calculated by averaging over periods when conductivity distribution in the region of interest is stable. We use the notation (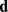) for time difference data and (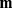) for the conductivity change image (or model) 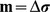. Depending on the image reconstruction approach, the image may be represented as a pixel grid, or on a finite element model (FEM) discretization. Of the many TD-EIT algorithms proposed (see references of [20], [29]) we consider only those that are applicable to the most common case for which clinical and experimental thoracic EIT measurements have been made. Thus, we assume: 1) TD-EIT measurements; 2) placement of 16 EIT electrodes in a single plane around the thorax; 3) measurement performed using adjacent stimulation and measurement (the Sheffield protocol); and 4) reconstruction of images onto a circular domain, since this is a common capability of all algorithms. We organize the space of EIT algorithms as in Fig. 2; first, algorithms are classified on the features used in the forward problem (and sensitivity matrix) and then on the inverse solution. Two main classes of image reconstruction procedures were chosen for analysis, based on backprojection, and sensitivity matrix (FEM) based solutions; the latter category is subdivided into regularization and optimization based methods. We review the formulation of each approach in the following subsections. Clearly, the possible combinations of algorithm variants are very large, and thus difficult to test. Instead, we define a "baseline" algorithm, 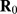 which represents the simplest approach, and consider modifications from it. For each variation, we define a default case (which is part of 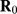) and a variant case, which is tested in an algorithm (Table 3).

**Table 3:** Algorithm variations considered. For each tested algorithm (horizontal row), the indicated variations from baseline (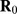) are made.

*Algorithm*	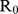	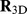	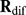	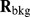	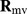	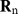	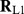	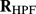	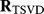	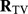	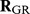	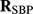
2D FEM (B)	√		√		√	√	√	√	√	√		
NOSER prior (B)	√	√	√	√	√	√						
3D FEM (F1)		√		√							√	
Diff. data (F2)			√									
Lung 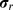 (F3)				√								
Elec. move. (F4)					√							
Noise weight (I1)						√						
Robust errors (I2)							√				√	
HPF prior (I3)								√				
TSVD (I4)									√			
Total variation (I5)										√		
Sheffield backproj												√

#### Backprojection sensitivity model

The backprojection algorithm was developed [43] for the original Sheffield EIT system, and has seen numerous variants and improvements. Using an analogy to X-ray CT backprojection, normalized measurements are "backprojected" onto the equipotential region in the image, and the image is subsequently filtered. Improvements to account for the diffuse nature of electrical current propagation in the body were subsequently made; a good mathematical characterization is given by Santosa and Vogelius [44]. While many variants of Sheffield backprojection (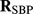) exist, clinical and experimental EIT has largely used only the one which was distributed in the Sheffield Mark I EIT system [45]. In discussions with the algorithm authors, it appears that the exact formulation of this algorithm has been lost. In order to accurately represent this algorithm, we obtained permission to reverse engineer 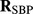 from a Sheffield Mk I device, as described in [29].

#### Numerical sensitivity model

The forward model, 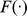, in EIT simulates the measured data vector 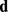 for a given vector of model parameters 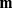, as 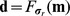. The data vector, 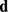, represents each measured voltage for each applied current pattern in a data frame; for the Sheffield stimulation protocol on 16 electrodes, this yields 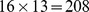 measurements per frame, of which 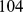 are independent, due to reciprocity [46]. 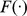 is typically based on a finite element model (FEM) and each parameter of the conductivity distribution is usually modelled in 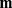 as the piecewise constant conductivity on a tetrahedral element. The forward model is used to calculate a Jacobian, 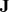, or sensitivity matrix, which describes the sensitivity of each data element to each model parameter. 
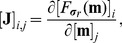
(1)where the FEM estimate depends on the background conductivity, 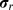, around which the conductivity changes 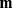 occur. The following section discusses various approaches to numerical (forward) models (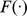), considering the baseline approach and its variants.

#### Model dimension: 2D FEM / 3D FEM

The choice of model dimension affects the relative magnitude of measured voltage in regions further from the current stimulations. Real voltage measurements occur in three dimensions, and are thus mismatched to 2D models. Early EIT algorithms used 2D FEM to reduce computational time; however, many recent algorithms continue to use 2D. One consequence is errors in the reconstructed position of objects because sensitivity falls off faster with distance in 3D than 2D.

**Figure 3:**
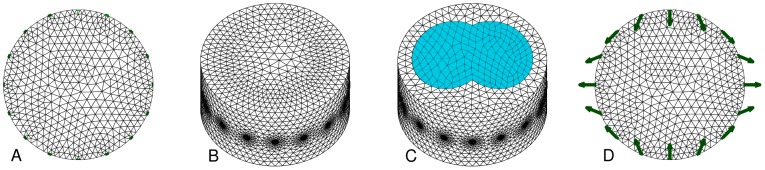
Finite element models used. Electrode nodes are indicated in green. A: 2D circular uniform FEM (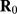) B: 3D cylindrical uniform FEM (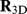) C: 3D cylindrical FEM with lung regions (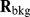) D: 2D circular uniform FEM with electrode movement (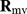) (with arrows showing representative electrode movement).

The baseline model uses a circular 2D FEM (Fig. 3A), and the variant uses a cylindrical 3D FEM of height equal to the medium diameter, and the electrode plane in the centre (Fig. 3B). FEM models are constructed using Netgen [47] by specifying the region geometry and circular electrode contact regions.

#### Difference data: normalized diff. data / diff. data

TD-EIT defines difference data 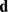 in terms of the current, 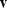, and reference, 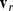 measurements. The majority of experimental and clinical algorithms (including 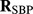) have used normalized difference imaging, where data 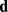 are defined as 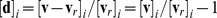. If difference imaging (without normalization) is used, then data 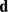 are defined as 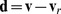. By normalizing, small measurement values are scaled up, and have a larger impact on the reconstructed images.

The baseline model uses normalized difference data, while the variant model uses difference data (without normalization).

#### Conductivity background: homogeneous 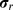 / lung 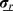


TD-EIT assumes conductivity changes occur with respect to a reference conductivity, 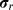, which defines the sensitivity matrix, 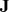. The most common choice for EIT algorithms is a homogeneous value for 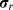, which is clearly a poor model for the thorax, in which the lungs are far less conductive than other tissues. The use of a homogeneous thorax conductivity distorts the position and amplitude of reconstructed contrasts [48].

The baseline model uses a homogeneous 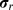 (Fig. 3B), while the variant model defines lung tissue with a relative conductivity of 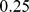 of that of other tissues [42]. The lung region is defined from CT images of pigs of similar size to those used in our experiments (Fig. 3C). We evaluate the effect of lung 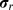 in a 3D FEM, since a full dimensional model is required to get more accurate current flow in the thorax.

#### Model electrode movement: static model / elec. move

One key difficulty with EIT measurements is due to the position uncertainty of the electrodes, especially for thoracic measurements, in which the body surface moves during breathing and posture change. Several approaches have been proposed to reduce the effect of electrode movements on the reconstructed EIT conductivity changes. We select the algorithm of [21], [49] based on using an augmented 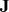, sensitive to impedance changes, electrode movement, and shape deformation in the model. This approach allows estimation of the shape changes, which we do not use here; instead, by allowing calculation of movement, certain artefacts in the conductivity image may be reduced. This approach can be shown to be equivalent to formulating electrode movement as a structured noise term which is added to the measurement noise variance, 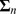. Following [49], 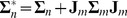
(2)where 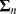 and 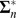 represent the original and updated estimates of data noise variance, respectively. 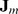 is the movement sensitivity matrix (change in data for each electrode coordinate change) and 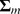 is a prior estimate of the covariance of electrode movement.

The baseline model assumes electrode position to be fixed, while the variant model updates noise estimates using (2).

#### Regularized EIT reconstruction

The class of linear regularized EIT reconstruction algorithms seeks a solution 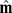 which minimizes the expression 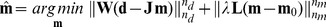
(3)


The first term, 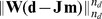 is the data error term, the mismatch between the model and the measured data weighted by a matrix 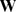, which models the measurement error on each data channel. The data error term has a norm 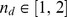. If a Gaussian model of data errors is used (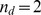), the data weighting matrix, 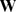, is related to the channel noise covariance, 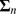, by 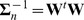. This formulation is also used to incorporate the structured noise due to electrode movement, 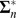 in (2). The second term, 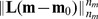 is a regularization term designed to address the ill-conditioning intrinsic to EIT; it serves as a penalty function which "discourages" unlikely (but otherwise feasible) solutions. It calculates the mismatch between the 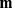 and prior constraints on "likely" models. 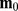 is the prior mean and has always been set to zero for TD-EIT, since conductivity is as likely to increase as decrease. 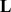 implements a penalty for large changes or non-smooth conductivities. The model error term has a norm 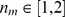. 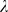 controls the relative strength of data and model error terms, and is typically called the regularization hyperparameter. As 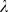 increases, the solution matches more closely to the model, and becomes increasingly smooth. In order to adequately compare different algorithms, functionally equivalent values of 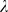 are chosen. Many strategies to choose an appropriate value have been presented (see references of [50]). We choose the "Noise Figure" approach [51], in which 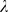 is chosen such that the ratio of values of signal to noise ratio (SNR) between 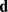 and 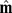 is required to be constant across algorithms. Since we have no control of the parameters of the SBP algorithm, all other algorithms are normalized to its value.

The most commonly studied case is for quadratic norms 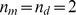, which has the advantage of modelling Gaussian error, and yielding a closed form, linear solution: 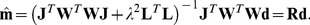
(4)


EIT algorithms of this form are known as one-step Gauss Newton solvers (GN) [52]. One important advantage is that image reconstruction can be expressed as matrix multiplication by 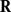; since 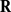 may be pre-calculated, reconstruction is rapid and may be implemented in real-time. If either 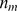 or 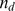 are not quadratic, image reconstruction must be formulated iteratively.

In general, regularized EIT image reconstruction techniques are popular due to their flexibility to represent arbitrary body geometry, measurement configurations, and mathematical models of the conductivity distribution. Many papers have used the basic regularized formulation, and have explored the choices of parameter values. In this paper, the baseline reconstruction algorithm, 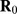, follows an early widely used regularized approach, NOSER [52], which makes the following parameter choices: it is quadratic, 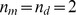, data noise is modelled to be identical on each channel 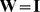, and the regularization matrix, 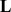, is diagonal such that, 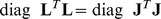, which implies an assumption that inter-element correlations are zero.

The following section discusses various approaches to regularized image reconstruction (I), considering the baseline approach and its variants.

#### Data noise model (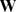): uniform / weighted

The most common approach is to "trust" all EIT data equally, and thus to model each channel with equal, uniform noise (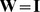). It is not necessary to set the actual noise intensity, since the data to model noise trade-off is set by the hyperparameter, 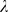. In practice, however, EIT noise can vary considerably between data channels, most likely due to variability in electrode contact quality. Such variations in data quality can be addressed by decreasing 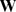 on channels with larger noise. One practical approach to setting an appropriate 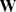 is to calculate the reciprocity error [53] (the difference between data values that should be equal by reciprocity [46]) and to use this value to scale 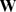. Clearly, modelling data noise would be expected to be more useful for lower quality data; we consider the EIT data in this study to be representative of relatively good measurements.

The baseline model sets 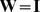, and the variant model sets 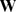 using the reciprocity error approach [53], setting the parameter 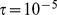. This parameter choice implied data were on average weighted at 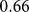 of their weighting in the baseline model.

#### Data norm (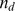): Gaussian, 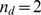 /robust, 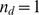


As mentioned, a Gaussian (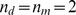) model and data norms allow calculation of a linear matrix inverse. One disadvantage of this formulation is that it emphasizes larger errors (since the effect of an error is squared). One approach to address such errors is the use of a 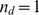 data norm. While such as approach has been relatively recently proposed in medical EIT [54], it has been widely used in the geophysical EIT literature, and is referred to as "robust error norms".

The baseline model uses a Gaussian data norm 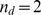, while the variant uses 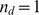 using the formulation of [54].

#### Prior model (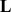): diagonal / smoothing

The regularization matrix, 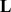, is chosen to penalize unlikely solutions. The NOSER [52] regularization matrix is diagonal, and thus penalizes large image amplitudes. Several studies have suggested that a better prior model is a smooth, rather than simply low amplitude distribution [51], [55], [56]. To achieve such smoothing, 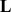 is designed to penalize non-smooth 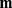, imposing a filter across nearby model elements. One key difference between an amplitude and a smoothing filter is its behaviour as a function of model element density. As density increases, amplitude penalties tend to show a more "speckled" pattern of noise, since each image element is independent in this case.

The baseline model sets 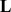 to penalize image amplitude [52], and the variant model uses the spatial high pass filter [51], in which 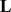 is calculated to apply Gaussian filter where spatial wavelengths below 10% of the medium diameter are penalized.

#### Prior model (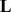): diagonal / truncated SVD

The function of regularization may be understood via the singular value decomposition (SVD) of the Jacobian, 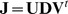, as the pseudo-inverse of 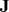, with the elimination of small singular values. Here, 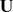 and 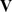 are orthonormal matrices and 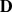 is diagonal. Thus 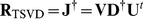
(5)where



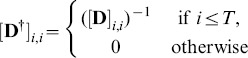
(6)The threshold 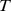 functions like a regularization parameter. We choose its value to enforce the noise figure constraint similar to the other algorithms. 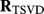 forms a linear inverse and may be represented in the form of (4) with the values 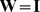 (uniform independent noise), a very large value of 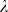, and 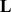 equal to 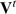 with its first 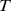 rows set to zero [57].

The baseline model sets 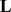 to penalize image amplitude [52], and the variant model uses the truncated SVD to set 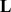 as indicated above.

#### Model norm (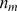): Gaussian / Total Variation

One disadvantage of a Gaussian (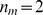) model norm is that it blurs edges in images, which is physiologically unrealistic, since organ boundaries are anatomically well-defined. This blurring can be addressed using the Total Variation (TV) formulation [58], with 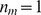. While TV produces apparently sharper images, there is some debate as to whether such images can give the appearance of features where none exist, especially in the context of EIT data errors.

The baseline model uses a Gaussian model 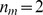, while the variant implements TV 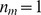 using the formulation of [54], [58].

#### Optimization based EIT reconstruction

One recent EIT reconstruction algorithm formulation is GREIT [29]. This algorithm proposes an approach to image reconstruction designed to implement a set of requirements (or figures of merit) on which a group of experts reached consensus. The parameters of a linear reconstruction matrix, 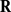, are chosen to best satisfy the listed requirements. This approach is similar in many ways to regularized image reconstruction; however it is the reconstruction matrix, 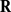, rather than the image, 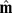, which is the target in the objective function formulation. Thus, we classify GREIT as an "optimization-based EIT reconstruction". The GREIT approach provides a number of different parameters which can be selected for an image; however, in this paper we choose the circular model reconstruction matrix evaluated in [29].

#### EIT algorithms evaluated

In order to test the large variety of possible combinations of EIT reconstruction algorithms, we evaluate a baseline algorithm, 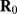, and modifications of it in a single variation. The list of considered algorithms is summarized in Table 3. We thus consider 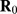 and nine variations, as well as SBP and GREIT, for a total of twelve algorithms.

### Data analysis

#### Image reconstruction

Each EIT algorithm identified in Table 3 was implemented using EIDORS version 3.6 [30] and used to reconstruct real conductivity change in each EIT frame in each series of EIT scans, representing 60 seconds of recording for each animal and condition. The reference data set, 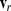 was chosen to be an average of data scans at end-expiration. Images reconstructed onto an FEM were interpolated onto a 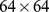 pixel grid (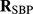 and 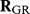 create a 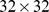 pixel image directly).

Modern algorithms can be tuned to control the trade-off between resolution and noise performance, generally via a "hyperparameter". To fairly compare algorithms, it is essential that the individual hyperparameters are set to equivalent values using an objective measure. Several such measures have been proposed [50]. In this study we chose to tune all algorithms such that the noise figure (NF) measure, first proposed for EIT by [51], for changes half the model radius away from the centre was 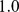. This particular value was chosen as it matched the performance of the backprojection algorithm, which does not have a tunable hyperparameter.

#### Ventilation pattern evaluation

From each sequence of EIT data, an average end-inspiratory and end-expiratory data scan was calculated and used for subsequent analysis. The average data scan was calculated by, first, identifying candidate end-inspiratory (end-expiratory) time points from the maximum (minimum) values in the average time course of EIT data. These candidate points were reviewed by a human operator in order to identify and, if necessary, reject physiologically infeasible time points. An example of identified (and rejected) events is shown in Fig. 4. The remaining points were averaged to create a single data scan, one for end-inspiration and one for end-expiration. Using these data, functional EIT scans were generated showing the distribution of local V_T_ by plotting the average tidal differences between the end-inspiratory and end-expiratory values in corresponding pixel locations.

Lung areas (A_L_) in images were identified as regions with an EIT ventilation signal above 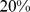 of the maximum found in the image. This approach was recommended e.g. in [59]. These were aggregated over all 6 recordings to provide a single lung region of interest (ROI) for each animal and algorithm. The size and shape of the lung ROI varies for different algorithms, since some tend to "squeeze" image contrasts toward the image centre. For each measurement, we calculate V_T_ by summing all pixel values within the lung ROI; we characterized the distribution of ventilation along the dorsoventral axis within the lung ROI as centre of ventilation (CoV), calculated analogously to centre of gravity, such that CoV 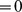 in the image centre and positive in non-dependent (ventral) lung regions; 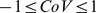. The CoV values depend on the position of the lung ROI within the EIT image. A decrease in CoV reflects a shift of ventilation distribution towards the dependent lung.

#### Statistical analysis

Statistical tests are performed as follows: to test for inequality (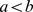), we use the one-tailed 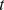-test to test rejection of the null hypothesis, 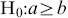; to test for equality (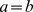), we use the two-tailed 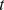-test to test rejection of the null hypothesis, 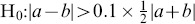 for V_T_, and 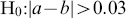 for CoV. Thus we consider EIT-derived V_T_ values equal when their means differ by less than 10%. We treat mean CoV values equal when they differ by less than 0.03, which corresponds to half a pixel height in a 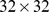 image. For each test a 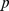 value is calculated, and 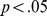 is considered significant.

The 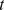-test requires an assumption of a normal distribution of data. We investigated this assumption using two approaches. First, the distribution of each set of calculated parameters was tested against the normal distribution using the Kolmogorov-Smirnov normality test, using 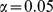. Next, data were analysed using the same protocol, but with the Wilcoxon signed rank test instead of the 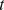-test.

## Results

**Figure 5:**
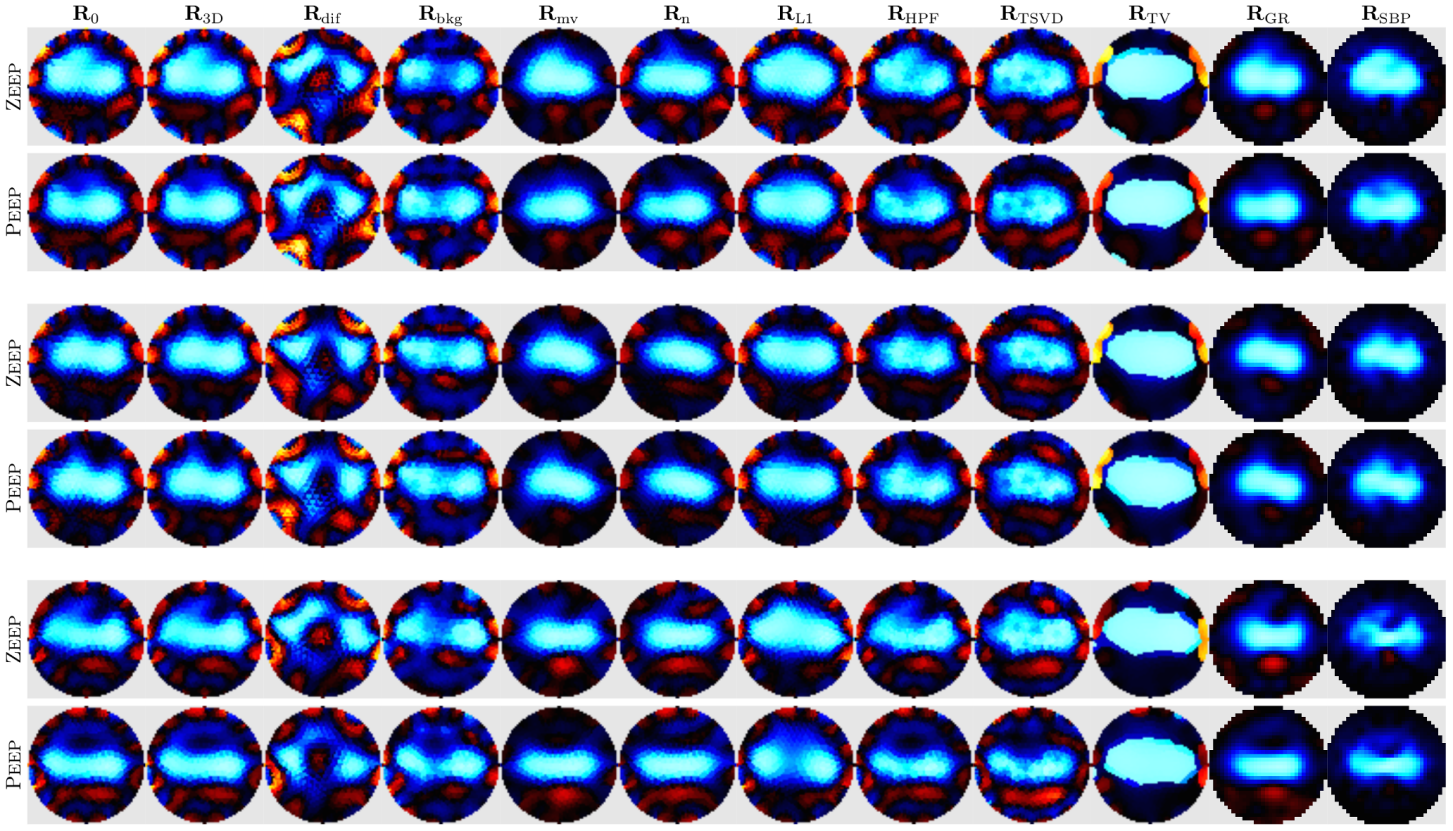
EIT images for all algorithms from three representative animals. For each animal, images of V_T_ at ZEEP and Peep are shown individually normalized to the maximum amplitude in each image (Blue: decrease in conductivity, Red: increase in conductivity). Each column shows images for a different algorithm. Peep, positive end-expiratory pressure; ZEEP, zero end-expiratory pressure; V_T_ tidal volume.

Images were reconstructed for all animals for each case and algorithm. Fig. 5 shows representative images of tidal change V_T_ at Peep and ZEEP in three animals under 21% F_I_ o_2_.

**Figure:**
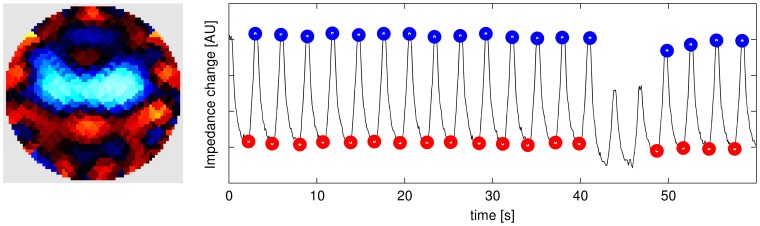
Sample image and identified end-inspiratory (blue) and end-expiratory (red) events. Left: Image of average tidal volume change Right: Average EIT signal (arbitrary units) vs time (s) showing identified events.

### Qualitative assessment

All algorithms successfully reconstructed the lungs as a central object with conductivity changing with ventilation (shown in blue as decrease in conductivity between expiration and inspiration). Images for different algorithms vary qualitatively in the shape of the lung region and the type and amount of artefacts in the images (Fig. 5). We qualitatively identify five types of artefacts: 1) boundary artefacts, especially near the electrodes, represented as image contrasts at the image edge; 2) speckle, showing high spatial frequency contrasts especially visible at the lung boundary (especially 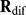, 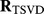); 3) ringing, showing inverse contrast near larger image contrasts (ie. red regions adjacent to blue lungs) (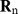, 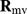, 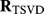); 4) spatial distortions, which disturb the shape, visible especially in the shape of the lung regions (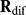); and 5) streak artefacts, showing contrasting lines radially projecting toward the electrodes (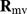, 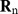, 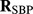). The presence of such artefacts disturbs the analysis of the images in several ways, primarily by introducing noise in the images, and by disturbing the analysis of the regional distribution of ventilation.

Images reconstructed with the baseline algorithm 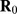 show the lungs as a single object. Although the images are generally smooth, boundaries of individual triangular finite elements on which the images are reconstructed prior to rasterization are clearly visible giving the appearance of implausible structures. This is true of all algorithms beside 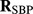 and 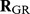 which reconstruct directly onto a pixel grid. Artefacts are clearly visible on the periphery near the electrodes (mostly in red).

Modifications of the numerical sensitivity model (algorithms 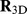 to 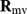) generally have an obvious and pronounced impact on the reconstructed EIT images. Only images obtained with 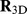, which differs from 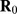 only in that a 3D forward model is used, do not show appreciable difference with respect to 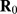. Reconstructions of difference data with 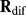, as opposed to normalized difference data as in 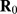, show much more artefacts than the other algorithms. However, although the lung shape appears distorted, it shows clear separation between the two lungs. The replacement of homogeneous 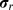 with one that includes lung tissue contrast in 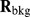 produces images with clearly separated lungs but slightly more artefacts than 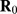. Reconstructions with electrode movement 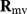 show virtually no boundary artefacts. However, the lungs appear slightly smaller and more round in comparison to 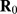.

Modifications of the parameters of regularized reconstruction also dramatically affect the appearance of EIT images. Noise weighting based on reciprocity in 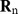 produced images with less artefacts than 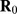, but more than 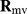. However, tidal changes seem to be pushed together and toward the centre resulting in comparatively smaller lungs. 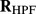 also offers limited reduction in boundary artefacts. In contrast, images reconstructed with 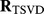 show increased artefacts compared with 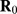; the lungs have a speckled appearance. As expected, 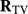 produced images with sharp boundaries. However, the resulting lung shape does not look plausible. Perhaps surprisingly, the use of the robust data norm with 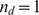 in 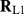 seems to offer no advantage in terms of boundary artefacts over 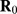 in our data set, but it reconstructs the lungs as bigger and, in some cases, better separated. Images produced with 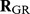 and 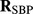 stand out for their smoothness and lack of boundary artefacts. 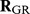 is most comparable with 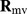 in terms of the shape of the lung region, although the lungs appear slightly smaller and less separable. Images obtained with 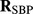 are similar to those obtained with 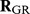, but the lungs appear even smaller while their shape is less smooth. It also seems to be distorted – tidal changes are pushed toward the centre and streak artefacts pointing outwards seem to show implausible structures.

### Functional assessment

The changes in ventilation pattern apparent in the reconstructed images were tested against physiological references, listed in Table 4, where 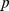 values of the corresponding statistical tests are also reported. The 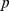-values are based on the 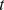-test, which assumes parameter values are normally distributed. We justify this assumption as follows. First, all parameter values pass the Kolmogorov-Smirnov normality test (at 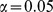). Second, we recalculated all 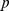 values using the Wilcoxon signed rank test, which showed substantially similar results; compared to the values in Table 4, the ordering of algorithms by 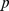-value was close to identical for all parameter values.

The independence of V_T_ from airway pressure was confirmed for most algorithms at F_I_ o_2_ of 100% (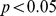 in Table 4). Notable exceptions are: 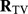, 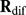 and, surprisingly, 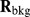. However, at F_I_ o_2_ of 21% V_T_ was less stable and only five algorithms passed the independence test (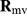, 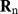, 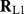, 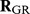 and 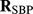). Nonetheless, V_T_ was independent of F_I_ o_2_ for all algorithms. Repeated measurements at the same settings showed very little variation in V_T_, which was reproducible for all algorithms.

At 21% F_I_ o_2_, no significant decrease in CoV associated with the introduction of Peep was detected, save by the 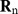 and 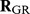 algorithms. In contrast, at 100% F_I_ o_2_, the distribution of ventilation was significantly more skewed towards ventral lung regions at ZEEP as compared to Peep. This is reflected in the decrease in CoV associated with the introduction of Peep, which was detected by all algorithms but 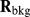 and 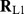 where it narrowly escaped significance. Accordingly, we found that the increase in F_I_ o_2_ from 21% to 100% produced for all algorithms a significant increase in CoV, i.e. a shift of ventilation distribution towards ventral regions, at ZEEP but not at Peep. Repeated EIT recordings with the same ventilator settings produced equivalent values of CoV for all algorithms at Peep. At ZEEP, CoV was reproducible at 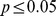 only for six algorithms (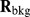, 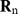, 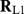, 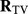, 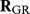 and 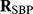), although only for 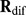 and 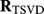 did 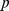 exceed 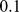.

## Discussion

EIT shows significant promise as a technique to monitor regional changes in lung ventilation, and to improve patient outcomes by informing ventilator therapy [4]. Since EIT is a challenging mathematical problem, many advanced reconstruction procedures have been developed, each offering potentially useful advances in terms of image quality or robustness against data noise. However, such advanced reconstruction schemes have seen little application to clinical or experimental EIT research, with the exception of GREIT [29] algorithm, which has seen some recent use.

In this paper, we propose a framework for functional validation and comparison of lung EIT images. This framework is then used to evaluate whether some advanced algorithms for EIT image reconstruction improve its ability to resolve the distribution of lung ventilation. As proposed, our validation focuses on EIT's ability to represent two key parameters, V_T_ and CoV, in accordance with known physiology. These are currently the most important for the proposed clinical applications of EIT; they have been applied in recent work by e.g. [60]–[62]. Additionally, our framework permits straightforward addition of new parameters by an extension of the evaluation software. Rather than using simulations or a saline phantom (e.g. [63]), our approach is more clinically orientated in that we use measures based on an in vivo experimental model. The key innovation is that we directly test reconstructed images against physiological "knowns". In particular, we test for the presence of significant changes in the reconstructed images as a result of experimental interventions with known physiological consequences. In the current study we do not assess the magnitude of these changes, although the presented framework could be extended in the future to include such assessment, once a number of technical difficulties are overcome. These include the use of normalized difference data, which hinders quantitative comparison of image pixel values to expected conductivity changes in experimental data; and the use of incorrect model geometry (discussed further below) resulting in spatially distorted images.

We consider algorithms based on a sensitivity model of EIT, which can be expressed in terms of a direct minimization of an error expression (eqn. 3). This includes many possible "ingredients": the dimension and background conductivity of the FEM; electrode movement modelling and reconstruction; time-difference vs. normalized time-difference measurement; data noise model; data and model norms; assumptions about the conductivity distribution imposed through the prior model; and method of calculating the pseudo-inverse of the sensitivity matrix. We do not consider all available algorithms; direct inversion methods (such as D-bar), those based on a Bayesian framework or non-linear sensitivity models (e.g. level set, monotonicity, or factorization) were not evaluated. We have designed our evaluation software to make it straightforward to add such comparisons as implementations of these algorithms become available. Even with the nine factors considered, it would not be possible to evaluate the full set of possible algorithms; instead we define a baseline algorithm and variants of it, yielding twelve algorithms, which were evaluated based on two approaches. First, representative images of ventilation were studied visually to assess the relative amount and character of artefacts as well as the shape of the region showing tidal changes. Second, measures of tidal changes and their distribution in the reconstructed images were tested against known physiological behaviour in experimental data.

To fairly compare algorithms we chose their hyperparameters, which control the trade-off between resolution and noise performance, such that the value of the noise figure (NF) metric [51] for a specific change in of 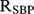. Still, because the NF of algorithms varies across the image, sometimes greatly, this approach to comparison is not completely fair for conductivity changes elsewhere in the domain. Moreover, this high value of NF means that for some of the tested algorithms the amount of regularization was insufficient to fully appreciate the nature of the prior.

Based on the results in Table 4, algorithms which exhibit a better 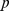-value are interpreted as offering an improved correspondence to known physiological "ground-truths", and are thus better. We do not select an overall "best", as that would amount to ranking the relative importance of the different tests, which is problematic. For example, the ability to accurately represent V_T_ may be more important relative to CoV in some clinical applications, but not others. Overall, we note that there is a general trend in which a ranking of algorithms by 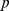-value performance in various tests are substantially consistent. Based on the images in Fig. 5, we make the following observations: 1) Artefacts on the image boundary are reduced in 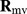, 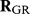 and 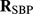, since these algorithms explicitly compensate for noise close to the electrodes; 2) The lung regions are better separated in 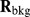 which exhibits increased sensitivity in the lung regions "pulling" them apart; 3) Reconstructions using difference – rather than normalized difference – data exhibit strong artefacts, likely due to the variation in gain on individual channels in the Goe-MF II EIT system; 4) Numerous "streak" artefacts are generated by 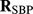, which are a result of wrong assumptions in the backprojection sensitivity model; 5) 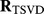, the only algorithm whose regularization is represented in terms of sensitivity matrix singular values – and thus not on the image space – produces strong "speckle" artefacts; 6) Algorithms using a spatial filter (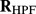 and 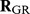) produce spatially smoother images; 7) On the other hand, 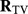 – designed to preserve boundaries – creates an image with implausible shapes; 8) The use of a 3D forward model, 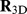 vs. 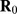, shows little appreciable difference despite the fact that EIT data from a 3D domain is incompatible with a 2D model [64], since the use of a circular shape is incorrect in both cases.

Overall, this means the variation in appearance of images reconstructed from the same data with different algorithms is not negligible. This poses a challenge to efforts to analyse the images at the level of individual pixels to derive diagnostic information. At the same time, the strength of our functional validation is evident in that we were able to detect clinically relevant physiological changes in spite of the varied appearance of the images. This is in contrast to methods based on comparison with images more faithful to anatomy that are primarily sensitive to morphological distortions.

Our results address two important questions:

1. *Do advanced image reconstruction algorithms provide advantages over the original Sheffield backprojection when applied to in vivo data?* Yes, some advanced algorithms offer a small, but significant advantage. Of the linear, one-step (i.e. fast) algorithms considered, 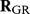 is the current best choice. Several other algorithms also offer advantages, such as those that consider electrode movement and the background lung conductivity; however, these "ingredients" can – and should – be included in an improved GREIT-like algorithm. Considering both the images and the significance values in our tests, the best approach (but at the cost of significantly higher computational complexity) is 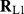, which uses robust error norms. We also note that several algorithms do not perform well.
2. *Do clinically relevant EIT measures characterizing lung ventilation depend on the algorithm used?* For ventilation therapy, EITs clinical relevance is driven by its ability to determine information on the changes in volume and its distribution, which we seek to capture in our evaluation framework. A first reading of Table 4 would indicate that the choice of algorithm affects the significance. Many algorithms have significantly worse 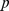-values than the original 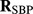, with which most clinical and experimental results have been analysed (ie. 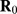, 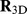, 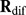, 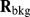, 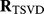, 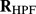, 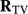). The remaining algorithms (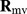, 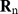, 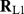, 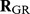) show similar, or in some cases improved, 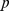-values to 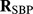. This group of algorithms may be characterized as "advanced in that they model additional image and sensor characteristics above that available in a classic GN regularized formulation. We note that the RSBP algorithm works surprisingly well given its vintage and ad-hoc formulation. Overall, our analysis does not suggest reconsideration of the validity of previous analyses of EIT data.

Our analysis is limited in that we only considered 2D electrode placement and adjacent injection and measurement patterns. These have prevented us from evaluating 3D reconstructions and limited the sensitivity in the centre of the image [49]. This, however, remains the de-facto standard for in vivo studies. We also purposefully used a circular (cylindrical in 3D) forward model for all algorithms, as this is what 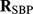 assumes, although using accurate body shape (e.g. derived from CT data) is known to decrease artefacts and improve positional accuracy [28], [65]. The effects of using a circular model do not affect our analysis of tidal volume changes, since V_T_ is a scalar not affected by the distribution of pixel values within the ROI used for its calculation. Furthermore, because CoV is a gross measure also defined on a ROI, it is unlikely that using an anatomically accurate model would substantially improve the performance of the evaluated algorithms in detecting shifts in CoV. However, the use of circular models is likely to have adversely affected our analysis of background modelling in 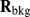, since realistic organ shape could not be incorporated into the forward model. Last but not least, our analysis considered the effect of each "ingredient" individually. Clearly, a combination of the more promising ones is likely to yield a better reconstruction algorithm than any of those tested here. However, naïve combinations are unlikely to produce good results. Instead, careful calibration and testing of many combinations will be needed, for which, we hope, the proposed evaluation framework will prove a useful tool.

## Conclusions

We present a methodology to experimentally evaluate lung EIT algorithms, and use it to analyse and compare recent work. The methodology is based on testing the algorithms ability to correctly detect physiological changes (or lack thereof) in a bespoke experimental data set. The basic idea behind this methodology allows it to be easily expanded to incorporate additional tests in the future. Several promising algorithm ingredients are identified; and we recommend research to evaluate approaches combining these factors, as well as other, non-linear algorithms. In order to support such investigation, we release the data and software for this study under a free license, so that it can be used and modified by others (http://sf.net/p/eidors3d/code/HEAD/tree/trunk/pubs/papers/compare-algs-2014).
